# Vaccine Induced Immune Thrombotic Thrombocytopenia After Sinopharm Vaccination: A Systematic Review of Case Reports

**DOI:** 10.1002/ccr3.70873

**Published:** 2025-09-10

**Authors:** Shafi Rehman, Mahnoor Sukaina, Hasibullah Aminpoor, Sissmol Davis, Amna Bint I Munir, Hasiba Karimi

**Affiliations:** ^1^ Department of Histopathology, Institute of Pathology and Diagnostic Medicine Khyber Medical University Peshawar Pakistan; ^2^ Karachi Medical and Dental College Karachi Pakistan; ^3^ Kabul University of Medical Sciences “Abu Ali Ibn Sina” Kabul Afghanistan; ^4^ JJM Medical College Davangere India; ^5^ Boston University Boston Massachusetts USA; ^6^ Bezmialem Vakif University, Faculty of Medicine Istanbul Turkey

**Keywords:** COVID‐19 vaccination, Sinopharm vaccine, thrombocytopenia, vaccine‐induced immune thrombotic thrombocytopenia

## Abstract

Vaccine‐induced thrombotic thrombocytopenia (VITT) has been linked to vector‐based vaccines, but only four cases following whole‐virus vaccines (Sinopharm) have been reported, as far as we are aware. This systematic review aimed to compile the clinical and laboratory results of VITT development after Sinopharm vaccination. We searched PubMed, ScienceDirect, and Google Scholar using pertinent keywords. The Preferred Reporting Items for Systematic Reviews and Meta‐Analyses (PRISMA) guidelines were followed when conducting the search. Inclusion criteria were human case studies, published in English, and freely available in full text. Of the 256 studies initially identified, 4 (1.56%) were analyzed. Mean age was 55.5 years (range: 18–85). There were *3 males* and *1 female* as per gender distribution. The most common symptom reported was sudden and extreme pain in the lower extremity (*n* = 2). The mean duration from the time of vaccination to admission was 7 days, and the average platelet count was 54.38 X 10^9^ /L. All (*n* = 4) cases had thrombocytopenia. VITT was definitively diagnosed according to ASH Criteria for VITT in 2 case reports. VITT adapted 4Ts scoring system showed high probability (8) in all (*n* = 4) cases. Intravenous immunoglobin (IVIG) was administered in 3 VITT patients, and all patients (*n* = 4) have successfully recovered. Early recognition and timely treatment improve patient prognosis.

**Summary**

- Vaccine‐induced immune thrombotic thrombocytopenia (VITT) is a rare but serious complication of COVID‐19 vaccination.
- This systematic review highlights four reported cases following Sinopharm vaccination. Early recognition, diagnosis using ASH criteria and 4Ts scoring, and timely treatment with nonheparin anticoagulants and IVIG can lead to favorable outcomes.
- Larger studies are needed.

## 1 Introduction

The coronavirus disease 2019 (COVID‐19) has been affecting people all around the world since December 2019. Vaccines are used all around the world to stop outbreaks and reduce mortality [1]. A range of immunizations is administered, such as vector‐based, whole‐virus, messenger ribonucleic acid (mRNA), and protein components [2]. Local injection site reactions are the most common adverse effects, followed by systemic symptoms such as fever, headache, fatigue, and myalgia. These side effects could develop shortly after immunization and go away fast [3].

Viral vector vaccines against COVID‐19 (Oxford‐AstraZeneca) (AZD1222 (ChAdOx1)) and Johnson & Johnson COVID‐19 (JNJ‐78436735 (Ad26.COV2S)) have been associated with thrombotic events and thrombocytopenia [4].

The term vaccine‐induced immune thrombotic thrombocytopenia (VITT) refers to this potentially fatal adverse effect [5, 6]. Sinopharm, an inactivated whole‐virus vaccine created by Chinese scientists, provides immunity against the coronavirus that causes severe acute respiratory syndrome (SARS‐CoV‐2) [7]. Tolerable adverse drug reactions, such as injection site reactions and fever, have been reported in prior immunization studies; however, no significant adverse reactions have been reported [8]. As of September 2023, four cases of VITT resulting from the inactivated whole‐virus vaccination (Sinopharm) have been reported. In this review, we analyzed reported cases of VITT following Sinopharm vaccination and discussed the diagnostic and therapeutic implications of their clinical and laboratory characteristics.

## 2 Materials and Methods

We conducted a comprehensive analysis of the existing literature on vaccine‐induced immune thrombotic thrombocytopenia (VITT) following Sinopharm immunization. The project did not require board approval because it met all the requirements for research using nonhuman subjects.

### 2.1 Search Strategy

The review adhered to PRISMA guidelines. Searches were conducted on Google Scholar, Science Direct, and PubMed. “Vaccine induced immune thrombotic thrombocytopenia” (VITT) AND “Sinopharm” (BIBP Vaccine) OR “BBIBP‐CorV Vaccine” OR “Covilo” were the MeSH words.

On September 13, 2023, the search was concluded. Table 1 illustrates the search approach. Only articles written in English and with human subjects were included in the results. After all abstracts were examined and the inclusion criteria were met, full‐text publications were obtained. Excluded were studies and publications that included insufficient or inaccurate information. Additionally, a manual search was conducted to find more relevant papers among the full‐text articles that were assessed later. The International Prospective Register of Systematic Reviews (PROSPERO‐CRD42023463257) was recruited and a protocol was registered.

**TABLE 1:** Search strategy for PubMed, Science Direct and Google Scholar.

Databases	Search strategy	Results
PubMed	(Vaccine induced immune thrombotic thrombocytopenia) OR (VITT) AND (((Sinopharm) OR (BIBP Vaccine)) OR (BBIBP‐CorV Vaccine)) OR (Covilo)	4
Science Direct	(“Vaccine induced immune thrombotic thrombocytopenia” OR “VITT”) AND (“Sinopharm” OR “BIBP Vaccine” OR “BBIBP‐CorV Vaccine” OR “Covilo”)	52
Google Scholar	(“Vaccine induced immune thrombotic thrombocytopenia” OR “VITT”) AND (“Sinopharm” OR “BIBP Vaccine” OR “BBIBP‐CorV Vaccine” OR “Covilo”)	1–200

### 2.2 Selection Criteria

We conducted a thorough search for observational studies with sizable sample sizes, but the results of our methodology were limited to case reports. Data on the diagnosis, course, treatment, and follow‐up of VITT following Sinopharm immunization were included in certain research. Animal research and non‐English language studies, research without full texts, research with little data, review studies, systematic reviews, and studies on VITT after any other COVID‐19 immunization were dropped, with the exception of the Sinopharm. Two reviewers (SR and MS) independently screened and analyzed studies, resolving discrepancies by discussion. Table 2 displays the case reports that were chosen using the Joanna Briggs Institute (JBI) Critical Appraisal Checklist for Case Reports, a quality appraisal tool. The original metrics for each parameter were combined with the data to undertake quantitative analysis.

**TABLE 2:** Quality assessment of case reports.

Authors	1	2	3	4	5	6	7	8
Devi et al. (2002)	Yes	Yes	Yes	Yes	Yes	Yes	Yes	Yes
Zaheri et al. (2002)	Yes	Yes	Yes	Yes	Yes	Yes	Yes	Yes
Sistanizad et al. (2023)	Yes	Yes	Yes	Yes	Yes	Yes	Yes	Yes
Hosseinzadeh et al. (2022)	Yes	Yes	Yes	Yes	Yes	Yes	Yes	Yes

*Note:* (1) Were the patient's demographic characteristics clearly described? (2) Was the patient's history clearly described and presented as a timeline? (3) Was the current clinical condition of the patient on presentation clearly described? (4) Were diagnostic tests or assessment methods and results clearly described? (5) Was the intervention or treatment procedure clearly described? (6) Was the postintervention clinical condition clearly described? (7) Was the adverse events or unanticipated events identified and described? (8) Does the case report provide takeaway lessons?

Abbreviation: JBI, Joanna Briggs Institute.

### 2.3 Data Extraction and Analysis

Variables included author, year of publication, study type, patient demographics, physical examination findings, laboratory investigation, treatment, and follow‐ups. Data analyses were performed with Microsoft Excel 2018 (Microsoft Corp., Redmond, WA, USA). The results are collated in Table 3 and Table 4.

**TABLE 3:** Summary of case report.

Authors/Year	Age	Gender	Country	Ethnicity	Chief complaints	Physical examination findings	Vaccine dose (1st or 2nd)	Time from vaccination to admission	Past medical Hx and medication	Past exposure of heparin	Historical platelet count prior to admission	Hx of allergy	Radiological investigation	Treatment	Follow‐up
Devi et al. (2002)	73	M	Pakistan	N/A	Acute chest pain	Left leg swelling along with tenderness.	N/A	14 days	Diabetes Mellitus	No	280 × 10^9^ /L.	N/A	DU: DVT of left popliteal vein; CT: B/L thrombosis in pulmonary veins.	IVC filters, Rivaroxaban	Alive and Stable
Zaheri et al. (2002)	46	W	Iran	Iran	Sudden and extreme pain in left calf, dyspnea, and chest pain	Painful, Pulselessnes, Pale, and hypothermic Calf	2nd dose	3 days	N/A	N/A	N/A	N/A	DU: thrombosis was found in the distal region of the left superficial femoral artery CTA: submassive emboli in the pulmonary artery	Bypass vascular surgery of, Dabigatran, IVIG	Alive and Stable
Sistanizad et al. (2023)	18	M	Iran	Caucasian	Severe pain and tenderness in the right arm.	Reduced power in lower limbs, Decreased DTRs	1st dose	10 days	N/A	N/A	N/A	N/A	CTA: infarct at subsegmental branches.	Apixaban, IVIG	Alive and Stable.
Hosseinzadeh et al. (2022)	85	M	Iran	Iran	Fever, chills, abdominal pain	LUQ tenderness.	N/A	1 day	IHD	N/A	337 × 10^9^/L	N/A	DU: thrombosis in the splenic vein with mild splenomegaly	Dexamethasone, IVIG and rivaroxaban	Alive and Stable

Abbreviations: B/L, bilateral; CT, computed tomography; CTA, computed tomography angiography; DU, doppler ultrasound; DVT, deep venous thrombosis; F, female; IHD, ischemic heart disease; IVC, inferior vena cava; IVIG: intravenous immunoglobulins; LUQ, left upper quadrant; M, male; N/A, not available/applicable.

**TABLE 4:** Laboratory characteristics of patient on admission.

	WBC (×10^9^ /L)	Platelet (×10^9^ /L)	Hemoglobin (× g/dL)	Anti‐PF4 antibodies (ELISA)	Gel agglutination assay of heparin/PF4 antibody (BIO‐RAD)	SARS COV‐2 antibody	PCR SARS COV‐2	D‐dimers (mg/dL)	ALT (U/L)	AST (U/L)	CPK (IU/L)	Fibrinogen (mg/dL)	FDP (μg/mL)	NT‐pro BNP (pg/mL)	PT (sec)	INR	PTT (sec)	LDH (IU/L)	CRP (mg/L)	ESR (mm/h)
Devi et al. (2002)	16.5	78	13	N/A	−ve	N/A	N/A	30	N/A	N/A	N/A	183	> 20	N/A	14	N/A	27	N/A	N/A	N/A
Zaheri et al. (2002)	17.1	67.5	12.9	+ve	N/A	N/A	−ve	67	N/A	N/A	N/A	206	33	N/A	16	N/A	30	N/A	N/A	N/A
Sistanizad et al. (2023)	4	57	13.0	N/A	N/A	N/A	−ve	2.82	59	173	5642	339	20.7	14,391	17.7	1.64	N/A	754	56	22
Hosseinzadeh et al. (2022)	2.8	15	9.4	+ve	N/A	+ve	+ve	3.2	N/A	N/A	N/A	172	N/A	N/A	N/A	′1	28	816	89	60

*Note:* Normal Values: WBC (4.5 to 11.0 × 10^9^/L); Platelets (150 to 400 × 10^9^/L); Hemoglobin (Male: 13.8 to 17.2 g/dL, Female: 12.1 to 15.1 g/dL), D‐dimers (< 0.50 mg/L of FEU), ALT (7–56 U/L), CPK (20–200 IU/L), Fibrinogen (200‐400 mg/dL), FDP (< 10 μg/mL), NT‐pro BNP (< 100 pg/mL), PT (11–13.5 s), PTT (25–35 s), INR (< 1.1), LDH (140–280 IU?L), CRP (< 8 mg/L), ESR (0‐20 mm/h).

Abbreviations: ALT, alanine aminotransferase; AST, aspartate aminotransferase; aPTT, activated partial thromboplastin time; CPK, creatine phosphokinase; CRP, C‐reactive protein; ESR, Erythrocyte Sedimentation Rate; FDP, fibrin degradation products; INR, International Normalized Ratio; LDH, lactate dehydrogenase; PT, prothrombin time.

## 3 Results

**FIGURE 1:**
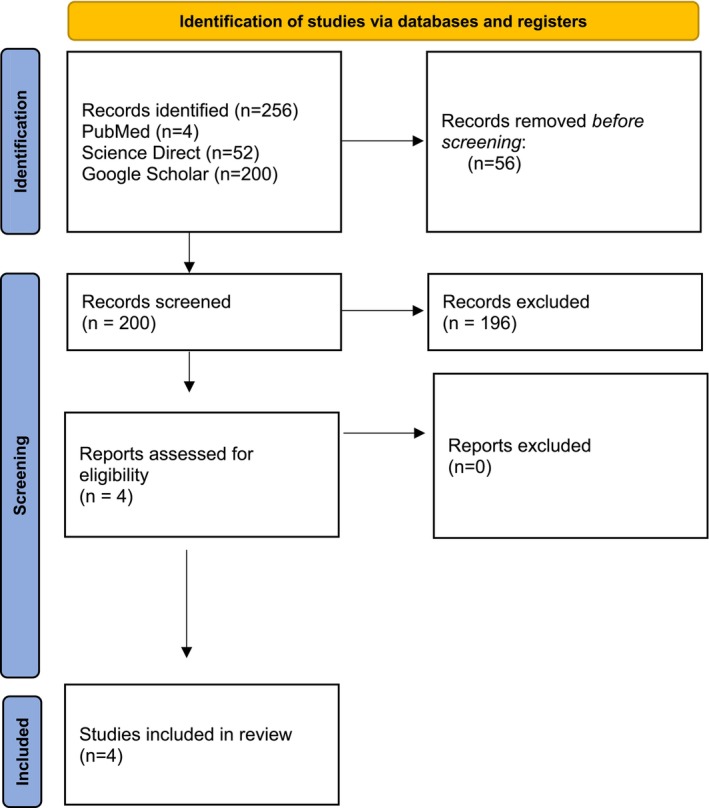
PRISMA 2020 flowchart. PRISMA, Preferred Reporting Items for Systematic Reviews and Meta‐Analyses.

A PubMed, ScienceDirect, and Google Scholar search was performed with 256 articles (Figure 1). A total of 4 case reports were included Devi et al. (2002), Zaheri et al. (2002), Sistanizad et al. (2023); Hosseinzadeh et al. (2022) were included in the analysis. The studies were from 2 different countries (Pakistan and Iran). The results are collated in Table 3 and Table 4. The ASH Criteria details are displayed in Table 5. The details of VITT adapted 4Ts scoring system are displayed in Table 6.

1. *Demographics*: Mean age at presentation was 55.5 years (range 18–85); There were three males and one female. Ethnicity was reported in 3 cases (2 Iranian, 1 Caucasian).
2. *Symptomatology*: Acute chest pain (*n* = 2), severe lower extremity pain (*n* = 2), and upper extremity pain (*n* = 1) were common. Symptoms followed the first dose in 1 case and the second dose in another.
3. *Pertinent physical examination findings*: The information was reported in all 4 case reports. Left leg swelling along with tenderness was reported in 1 case, painful, pulseless, pale, and hypothermic calf in 1 case, and reduced power in the lower limbs with reduced deep tendon reflexes in the lower extremity in 1 case.
4. *Time from vaccination to admission*: Mean interval from vaccination to hospital admission was 7 days.
5. *Past medical history/Medication*: Diabetes mellitus (*n* = 1) and ischemic heart disease on aspirin (*n* = 1) were reported. Only the patient with ischemic heart disease was reported to be taking aspirin 80 mg.
6. *Past exposure to heparin*: None of the patients had prior heparin exposure.
7. *Historical platelet count prior to admission*: Preadmission platelet count was available in 2 case reports; both were normal prior to admission.
8. *History of allergy*: None reported.
9. *Radiological investigation*: The information was available in all 4 case reports. Radiological investigations revealed deep venous thrombosis of the left popliteal vein (1 case), bilateral thrombosis in pulmonary veins (1 case), complete thrombosis in the distal region of the left superficial femoral artery (1 case), submassive emboli in the pulmonary artery (1 case), and thrombosis in the splenic vein with mild splenomegaly (1 case).
10. *Laboratory investigation*: The mean white blood cell (WBC) count was 10.1 × 10^9^/L, with two patients presenting leukocytosis and two with leukopenia. Platelet counts averaged 54.38 × 10^9^/L, and all patients exhibited thrombocytopenia. Hemoglobin levels averaged 12 g/dL, with no cases of anemia. Anti‐PF4 ELISA was positive in both patients tested, while one BIO‐RAD assay was negative. D‐dimer levels were markedly elevated, with a mean of 25.7 mg/L. Fibrinogen averaged 225 mg/dL, and fibrin degradation products (FDP) were elevated with a mean of 24.6 μg/mL in three cases. Additional laboratory abnormalities included elevated liver enzymes (ALT 59 U/L, AST 173 U/L), creatine phosphokinase (CPK 5642 IU/L), lactate dehydrogenase (LDH mean 785 IU/L), C‐reactive protein (CRP mean 72.5 mg/L), and erythrocyte sedimentation rate (ESR mean 41 mm/h). SARS‐CoV‐2 serology was positive for both spike and nucleocapsid antibodies in one case, and PCR was positive in another. Coagulation profiles showed a mean prothrombin time (PT) of 15.9 s (three cases), mean INR of 1.32 (two cases), and mean aPTT of 28.3 s (three cases).
11. *ASH criteria for VITT*: VITT was definitively diagnosed according to ASH criteria in 2 cases. In 2 other cases, VITT was not a definitive diagnosis due to the absence of anti‐PF4 antibody positivity by ELISA though all other parameters were fulfilled. The details are displayed in Table 5.
12. *VITT adapted 4Ts scoring system*: The VITT‐adapted 4Ts scoring system showed a high probability (score of 8) in all 4 cases. The details are displayed in Table 6.
13. *Surgical treatment*: Surgical treatment included the placement of an inferior vena cava (IVC) filter in 1 case and emergency bypass vascular surgery of the popliteal and femoral arteries in 1 case.
14. *Medical treatment*: Medical treatment included Rivaroxaban (2 cases), Dabigatran (1 case), intravenous immunoglobulin (IVIG) (3 cases), fibrinolytic and nonheparin agents (1 case), Apixaban (1 case), and intravenous dexamethasone (1 case).
15. *Follow‐up*: There were no reported relapses after treatment, and all four patients successfully recovered.

**TABLE 5:** ASH Criteria for diagnosing vaccine induced immune thrombocytopenia (VITT).

Authors	Covid vaccination^a^	Arterial/Venous thrombosis^b^	Thrombocytopenia^c^	Anti‐PF4 antibodies^d^	D‐dimers^e^
Devi et al. (2002)	Yes	Yes	Yes	No	Yes
Zaheri et al. (2002)	Yes	Yes	Yes	Yes	Yes
Sistanizad et al. (2023)	Yes	Yes	Yes	No	Yes
Hosseinzadeh et al. (2022)	Yes	Yes	Yes	Yes	Yes

^a^ COVID vaccine 4 to 42 days prior to symptom onset.

^b^ Any venous or arterial thrombosis (often cerebral or abdominal).

^c^ Thrombocytopenia (platelet count < 150 × 10^9^/L).

^d^ Positive PF4 “HIT” (heparin‐induced thrombocytopenia) ELISA.

^e^ Markedly elevated D‐dimer (> 4 times upper limit of normal).

**TABLE 6:** VITT adapted 4Ts scoring system.

Authors	Thrombocytopenia^a^	Timing^b^	Thrombosis^c^	Other cause of thrombosis or thrombocytopenia^d^	Interpretation^e^
Devi et al. (2002)	2	2	2	2	8 (High probability)
Zaheri et al. (2002)	2	2	2	2	8 (High probability)
Sistanizad et al. (2023)	2	2	2	2	8 (High probability)
Hosseinzadeh et al. (2022)	2	2	2	2	8 (High probability)

^a^ Platelet count 10,000 to 99,000/microL (2); Platelet count < 10,000/microL or 100,000 to 149,000/microL (1); Platelet count ≥ 150,000/microL (0).

^b^ 5 to 14 days post vaccine (2); 15 to 30 days post vaccine (1); 0 to 4 days or ≥ 30 days post vaccine (0).

^c^ Definite thrombosis or D‐dimer > 10 mg/L (> 10,000 ng/mL) (2); Suspected (not documented) thrombosis or D‐dimer 2.00 to 9.99 mg/L (2000 to 9990 ng/mL) (1); No thrombosis and D‐dimer < 2 mg/L (< 2000 ng/mL) (0).

^d^ None apparent (2); Possible (1); Definite (0).

^e^ 0 to 3 points—Low probability; 4 to 5 points—Intermediate probability; 6 to 8 points—High probability.

## 4 Discussion

### 4.1 History and Clinical Characteristics of Reported Cases

The history of vaccinations does not include a novel instance of thrombotic thrombocytopenia after immunization [9]. In 1973, Brown et al. reported thrombotic thrombocytopenia as a result of influenza vaccination [10]. Similar findings have been reported with pneumococcal, rabies, and H1N1 vaccinations in other studies [11, 12, 13, 14]. In multiple cases, rituximab, plasmapheresis, and corticosteroids were suggested as alternative therapies [10, 13].

The condition known as SARS‐CoV‐2 vaccine‐induced immune thrombotic thrombocytopenia (VITT) has been recently observed in the context of inactivated whole‐virus vaccination (Sinopharm) [15, 16, 17, 18]. According to a systematic review by Dorche et al. (2021) [4], all reported cases of VITT occurred following vaccination with AstraZeneca (ChAdOx1) and Johnson & Johnson (Ad26.COV2). Only 20% of VITT cases were documented in men, while 80% were in women. However, our data indicate that after receiving the inactivated whole‐virus vaccine (Sinopharm), 75% of male patients and 25% of female patients developed VITT.

Dorche et al. (2021) [4] reported that symptoms typically appeared 1 week after the first immunization dose (range: 4–19 days). This aligns with our review, where the average time from Sinopharm immunization to hospital admission was 7 days.

According to Dorche et al. (2021) [4], headaches were the most common presenting symptom in individuals with cerebral venous sinus thrombosis (CVST). However, our review found that the most frequent symptom was sudden, severe lower extremity pain. Additionally, Dorche et al. (2021) [4] reported that four individuals experienced middle cerebral artery (MCA) infarction following AstraZeneca (ChAdOx1 nCoV‐19) vaccination. Other described sites of thrombosis in their study included ischemic bowel infarction, splanchnic vein thrombosis, lower extremity deep vein thrombosis, bilateral adrenal hemorrhage, portal vein thrombosis, iliofemoral vein thrombosis, and internal jugular vein thrombosis. In contrast, thrombosis in our review was observed in the pulmonary, splenic, and upper and lower limb vasculature.

The platelet count in our study ranged from 15 to 78 × 10^9^/L, with a mean of 54.38 × 10^9^/L, whereas Dorche et al. (2021) [4] reported a platelet count range of 5 to 127 × 10^9^/L. Additionally, at least 21 (38.8%) of the 54 patients examined by Dorche et al. (2021) [4] succumbed to the condition, including 19 cases of CVST (39%), one case of infarction (25%), and one case of intracerebral hemorrhage (ICH). However, no mortality was documented in our review.

Given our small sample size of only four patients, we cannot make definitive conclusions about the safety of VITT after Sinopharm vaccination. Nevertheless, the absence of fatalities in our cohort is encouraging and may suggest that VITT following Sinopharm vaccination is less life‐threatening compared to other COVID‐19 vaccines. Future large‐scale studies are necessary to provide conclusive answers.

It is crucial to promptly diagnose and manage suspected cases of VITT following administration of any COVID‐19 vaccine, including Sinopharm, to ensure timely intervention and improved patient outcomes.

### 4.2 Pathophysiology

Clinically, vaccine‐induced immune thrombotic thrombocytopenia (VITT) resembles autoimmune thrombocytopenia induced by heparin, known as heparin‐induced thrombocytopenia (HIT). HIT is caused by the formation of complexes between heparin and platelet‐activating immunoglobulin G (IgG) antibodies against platelet factor 4 (PF4). Upon binding to platelet FcγRIIA receptors, this complex activates platelets, leading to the formation of platelet microparticles [19]. These microparticles promote clot formation, initiating a prothrombotic cascade that decreases platelet counts and results in thrombocytopenia. Moreover, antibody‐coated platelets are removed by the reticuloendothelial system, further exacerbating thrombocytopenia [3, 19, 20, 21].

Additionally, some patients exhibit clinical symptoms and laboratory features of HIT despite never having been exposed to heparin; these cases are classified as spontaneous autoimmune HIT. In these individuals, antibodies in their sera strongly stimulate platelets even in the absence of heparin. Most patients with confirmed spontaneous HIT have a history of orthopedic surgery, possibly due to infection‐related microorganism exposure or the release of glycosaminoglycans and RNA from tourniquet‐induced cell damage [22].

The development of VITT may be facilitated by interactions between the vaccine and platelets or PF4. This phenomenon may be explained by PF4 could electrostatically bind to the viral particles in VITT and trigger PF4‐reactive autoantibodies [23].

### 4.3 Diagnosis

The following American Society of Hematology (ASH) diagnostic criteria for vaccine‐induced immune thrombotic thrombocytopenia (VITT) following Sinopharm COVID‐19 immunization should be applied for a definitive diagnosis of VITT:

1. The patient received the vaccination within the previous 30 days (between 4 and 30 days).
2. Mild to severe thrombocytopenia is present. However, thrombocytopenia may be mild in some cases, particularly in the early stages of VITT.
3. The presence of thrombosis, which typically manifests as splanchnic vein thrombosis, causes back or abdominal pain, nausea, and vomiting. Arterial thrombosis is a very rare complication.
4. Anti‐PF4 antibody positive by ELISA.

Although transient headaches are a common postvaccination side effect, VITT should be considered if a patient presents with persistent headache, blurred vision, petechiae, easy bruising, or bleeding following Sinopharm immunization [24, 25].

For a definitive diagnosis of VITT following Sinopharm vaccination, all four ASH criteria must be met. However, cases reported by Devi et al. (2022) and Sistanizad et al. (2023) did not fulfill all four ASH criteria due to the unavailability of facilities for ELISA testing of antibodies to the heparin‐platelet factor 4 complex. In such instances, the VITT‐adapted 4Ts scoring system [26], as shown in Table 6, can be utilized.

The VITT‐adapted 4Ts scoring system evaluates:

1. The degree of thrombocytopenia (0–2).
2. The time interval since vaccination (0–2).
3. Thrombosis status (definite, suspected, absent, and D‐dimer levels, 0–2).
4. Additional potential causes of thrombosis or thrombocytopenia (0–2).

A cumulative score of 6 to 8 indicates a high probability of VITT. In all reported cases (*n* = 4), the VITT‐adapted 4Ts scoring system suggested a high likelihood of VITT, with a score of 8.

### 4.4 Recommended Evaluations and Laboratory Workups

Laboratory workups should include a complete blood count (CBC) and peripheral blood smear, fibrinogen and D‐dimer levels, prothrombin time (PT), partial thromboplastin time (PTT), and PF4‐ELISA (HIT assay) [27]. The details of the laboratory tests are collated in Table 4, Table 5, and Table 6.

### 4.5 Treatment Strategies

Until VITT is ruled out, patients should refrain from using heparin [24]. Close collaboration among vascular surgeons, vascular neurologists, hematologists, and other professionals with relevant expertise is essential for managing VITT‐associated systemic thrombosis [27]. Despite the limited data on available therapeutic options, it has been advised to administer intravenous immunoglobulin (IVIG) at a dose of 1 g/kg body weight daily for 2 days following the shipment of PF4 antibodies [27]. IVIG blocks platelet FcγRIIA receptors, thereby preventing antibody‐mediated platelet clearance and potentially inhibiting platelet activation [21].

Some experts recommend high‐dose glucocorticoids, which may improve platelet counts within a few days [21]. Another potential treatment option for correcting coagulopathy and reducing abnormal antibodies is plasmapheresis [28]. However, platelet transfusions should be avoided due to the risk of further antibody‐mediated platelet activation and worsening coagulopathy [28].

Therapeutic anticoagulation [23] can be achieved using nonheparin anticoagulants, including direct oral factor Xa inhibitors (Apixaban, Rivaroxaban), direct thrombin inhibitors (Argatroban, Bivalirudin), and indirect (antithrombin‐dependent) factor Xa inhibitors such as Fondaparinux and Danaparoid. In cases of low fibrinogen levels or severe thrombocytopenia (less than 20,000/mm^3^), the dosage strategy may need adjustment [27]. For critically ill patients, parenteral medications with short half‐lives are preferred [23, 27]. Even in cases of secondary intracranial hemorrhage (ICH), anticoagulation should be administered during cerebral venous sinus thrombosis (CVST) to prevent further thrombosis [27]. Once complete platelet count recovery is achieved and no contraindications remain, direct oral anticoagulants or vitamin K antagonists are recommended for subacute or chronic therapy [27].

## 5 Risks and Benefits of Vaccination

Patients with COVID‐19 face a 1% to 2% mortality risk, along with several long‐term complications [29, 30]. Individuals with preexisting medical conditions or those on specific medications may be at significantly higher risk [31]. Conversely, vaccination has a very low incidence of serious adverse effects and plays a crucial role in preventing severe SARS‐CoV‐2 infection and its associated complications [32]. The estimated incidence of VITT is approximately one case per 100,000 vaccine exposures [21].

Therefore, as the benefits of COVID‐19 immunization outweigh the risks, vaccination should be strongly recommended [21]. However, health authorities must continue monitoring adverse vaccine effects to ensure public safety.

## 6 Limitation

There are currently only a small number of case reports of VITT after Sinopharm immunization, which limits the current systematic study. Major observational studies that provide trustworthy data are nonexistent. Because case reports are not intended to have internal validity, any conclusions drawn from them need to be supported by observational studies and clinical trials, which were not possible with a sample size of only four case reports (*n* = 4). The limited sample size and significant patient data heterogeneity precluded us from inferring a causal relationship between Sinopharm immunization and the onset of VITT.

## 7 Conclusion

The clinical signs, etiology, diagnostic standards, and treatment approaches for VITT issues after Sinopharm covid‐19 vaccination are all included in this review. Early diagnosis and appropriate treatment planning may improve patient outcomes. The relationship between VITT and Sinopharm immunization requires more investigation.

## Author Contributions

**Shafi Rehman:** conceptualization, data curation, methodology, project administration, supervision, writing – original draft, writing – review and editing. **Mahnoor Sukaina:** conceptualization, methodology, project administration, writing – original draft, writing – review and editing. **Hasibullah Aminpoor:** data curation, writing – original draft, writing – review and editing. **Sissmol Davis:** data curation, writing – original draft, writing – review and editing. **Amna Bint I Munir:** data curation, writing – original draft, writing – review and editing. **Hasiba Karimi:** data curation, writing – original draft, writing – review and editing.

## Ethics Statement

The authors have nothing to report.

## Consent

Written informed consent was obtained from the patient. To publish this report in accordance with the journal's patient consent policy.

## Conflicts of Interest

The authors declare no conflicts of interest.

## Data Availability

Data sharing not applicable to this article as no datasets were generated or analyzed during the current study.
